# Exploring media framing of abortion content on Kenyan television: a qualitative study protocol

**DOI:** 10.1186/s12978-021-01071-5

**Published:** 2021-01-19

**Authors:** Catherine Kafu, Dina Ligaga, Juddy Wachira

**Affiliations:** 1 Department of Media Studies, Faculty of Humanities, School of Literature, Language and Media, University of the Witwatersrand, Johannesburg, South Africa; 2 Academic Model Providing Access to Healthcare (AMPATH) Partnership, Eldoret, Kenya; 3 Department of Behavioral Sciences, School of Medicine, Moi University, Eldoret, Kenya

**Keywords:** Media framing, Abortion, Adolescents, Sexual and reproductive health and rights, Journalism

## Abstract

**Background:**

Media framing of abortion messages is an emerging field of research. However, little is known about how the news media frames abortion messages aimed at influencing adolescents’ reproductive health choices. This study therefore seeks to investigate the framing of abortion in TV news items on three leading Kenyan TV outlets over a period of 3 years, understand Kenyan journalists’ perceptions and experiences with abortion coverage, and to examine adolescents’ perceptions and experiences with abortion coverage on Kenyan televised news media.

**Methods:**

This qualitative study which will be conducted in two sites-Nairobi and Uasin Gishu counties-in Kenya will purposively sample abortion news items from three leading media outlets aired between January 2016 to December 2019, for content analysis. Additionally, 12 journalists (9 reporters, 3 news editors) will be purposively sampled for Key Informant Interviews (KIIs) on journalist framing of abortion messages. Finally, convenience sampling will be used to select approximately 48 university-going adolescents for four Focus Group Discussions (FGDs)-2 female, 2 male- aimed at examining adolescents’ perceptions and experiences with abortion coverage in the broadcast news media. The KIIs and FGDs will be audio-recorded, transcribed and translated. These data will be analyzed thematically.

**Discussion:**

This study moves beyond interrogating only media items to further exploring framing from the perspectives of media consumers and investigations in the process behind production of abortion messages. The study interrogates abortion messages aimed at younger demographics such as adolescents as well as the gendered differences of the effects of these abortion messages, an area barely explored. The study findings will be informative to those who wish to develop media that could be used to promote safe abortion as well as advocate for sexual reproductive health rights, especially among adolescents.

## Plain English summary

Unsafe abortion continues to be a public health issue resulting to high morbidity and mortality rates especially among adolescents. Media has a great potential of supporting adolescents realize optimal Sexual Reproductive Health and Rights (SRHR). Media presents a great platform for creating awareness and motivating discussions about ASRHR issues especially around taboo topics like abortion. This builds an enabling environment where adolescents have access to information and services, as well as the capacity to make decisions on their own within the context of informed and supportive communities. However, the success of mass media interventions largely depends on the framing of the messages. To understand media’s role in the promotion or erosion of ASRHR, this qualitative study strives to investigate the framing of abortion in TV news items by conducting qualitative content analysis on news media items of three leading Kenyan TV outlets over a period of 3 years. Additionally, the study seeks to examine the implications of this framing on audiences by conducting Focus Group Discussions (FGDs) among adolescents’ (18–24 years) to understand their experiences and perceptions of news media’s framing of abortion. Finally, in an effort to understand the frame production process, Key Informant Interviews (KIIs) will be conducted among Kenyan journalists to explore their perceptions and experiences with abortion framing in televised news media. The study findings will be informative to those who wish to develop media that could be used to promote safe abortion as well as advocate for sexual reproductive health rights, especially among adolescents. Furthermore, the study will lay groundwork for future studies in news media framing of abortion messages by moving beyond the traditional approach of only interrogating media items to further exploring framing from the perspectives of media consumers and investigations in the process behind production of abortion messages.

## Background

There is growing research interest in the field of media coverage of abortion. Documented literature is predominantly quantitative and majorly conducted in the USA with a larger emphasize on adult women [1]. Little academic attention has been directed at examining media framing of abortion messages aimed at younger demographics such as adolescents [2] as well as the gendered differences of the effects of abortion coverage [3]. Despite the limited empirical attention to abortion in the media, unsafe abortion continues to be a public health issue resulting to high morbidity and mortality rates especially among adolescents [4]. This is especially true for Sub-Saharan Africa (SSA) where an estimated 2–5 million adolescents undergo unsafe abortion annually [5].

The Kenyan legal and socio-cultural contexts have been found to significantly contribute to unsafe abortions. Legality of abortion is an issue mired with confusion and contention in Kenya. Women and health care providers are not sure whether they are protected by the new constitution if they sought or provided abortion care services respectively [6]. This legal ambiguity provides a basis for the continued harassment, arrests and penalization of providers and women suspected of offering and seeking abortion services respectively, by both the public and government agencies [4, 7]. As a result, qualified health care providers have been pushed into operating clandestinely and charging exorbitantly. These charges are normally out of reach for majority of Kenyan women who then resort to cheap but unsafe abortion methods [8]. This is especially true for adolescents and women living in rural settings due to their limited economic power [9].

Moreover, teenage pregnancy and abortion are condemned and stigmatized in Kenya [10]. Teenage pregnancy is viewed as defiance of norms of sexual abstinence and propriety [4, 9]. While abortion is viewed as a violation of the ideals of femininity, adolescence, and womanhood [4]. Adolescents who seek to terminate a pregnancy challenge the ideals of womanhood that originate in conservative gender roles and intend to control female sexuality [8, 9]. The largely negative societal attitudes towards teenage pregnancies and abortion compounded by uncertainty about abortion law and perceived higher cost of safe providers force many adolescents to seek abortion unsafely, often with negative sequelae [7].

Determining the index of abortion is difficult due to the socio-cultural and legal issues involved, making it challenging to collect data. However, a study carried out between 2012 and 2016 by the African Population and Health Research Centre (APHRC), the Ministry of Health and Ipas revealed that approximately 360 women procure abortion daily with 77% of these abortions being unsafe. Approximately 21,000 women are hospitalized each year due to abortion related complications stemming from unsafe abortions [11]. An estimated 2600 women die from botched abortions annually [12]. Many more die at home without seeking medical care. Unsafe abortions account for 35% of maternal deaths in Kenya, much higher than the global average of 13% [13]. It further consumes a substantial amount of scarce health systems resources, [14] with an estimated KES. 533 million spent annually on treating complications from back-alley abortions [15]. These points to the need for continued work aimed at addressing adolescent sexual reproductive health and rights (ASRHR).

Media has a great potential of supporting adolescents realize optimal Sexual Reproductive Health and Rights (SRHR). Media presents a great platform for creating awareness and motivating discussions about ASRHR issues especially around taboo topics like abortion [16]. This builds an enabling environment where adolescents have access to information and services, as well as the capacity to make decisions on their own within the context of informed and supportive communities [16, 17]. However, the success of mass media interventions largely depends on the framing of the messages. To understand media’s role in the promotion or erosion of ASRHR, one needs to study how these issues are framed in the media and the implications of this framing on the audiences [18]. Furthermore, one needs to strive to understand the frame production process by acknowledging the role of news organizations and journalists in the framing of abortion messages [19], an area not well developed in literature. This study therefore seeks to:

- *Objective 1* Investigate the framing of abortion in TV news items on three leading Kenyan TV outlets over a period of 3 years
- *Objective 2* Explore Kenyan journalists’ perceptions and experiences with abortion framing in televised news media.
- *Objective 3* Examine adolescents’ (18-24 years) experiences and perceptions of media’s framing of abortion on Kenyan televised news media.

## Methods

This qualitative study will be conducted across two sites in Kenya. The study will apply qualitative content analysis (QCA), Key Informant Interviews (KIIs) and Focus Group Discussions (FGDs) to interrogate media items, investigate the process behind production of abortion messages and explore framing from the perspectives of media consumers. Due to the diversity of the methods in this study, we present the methods based on each objective.

### Objective 1: Investigate the framing of abortion in TV news items on three leading Kenyan TV outlets over a period of 3 years

#### Study site

This study will be conducted in Nairobi, which is the capital city of Kenya and the largest city in the country. It is home to most local news media organizations as well as the regional headquarters to several multinational media organizations. The three media outlets targeted by this study are located in Nairobi.

#### Sampling

The sampling unit for this study will be televised abortion news items aired Citizen TV, NTV and KTN news outlets. These television stations have been selected based on their popularity among Kenyan audiences [20]. The inclusion criteria will be:, news items must contain abortion related content with abortion being the focus of the news item, the news item addresses the Kenyan context, have been aired between January 1, 2016 and December 31, 2019 and must have been aired on either CITIZEN TV, NTV or KTN. Any social announcements, advertisements, news items merely mentioning abortion while focusing on other topics or duplicate abortion coverage will be excluded.

#### Data collection

A checklist (Table 1) based on the inclusion/exclusion criteria aforementioned will be used to determine the news items that qualify to be included in this study. Abortion news items will be retrieved from the CITIZEN TV, KTN and NTV digital archives based at the respective television stations headquarters in Nairobi. This will be achieved by searching the digital archives of these three television networks for abortion coverage. Individual archives search engines will be used to retrieve abortion news item the period from January 2016 to December 2019 employing the keywords “abortion OR abort OR termination of pregnancy OR pregnancy termination.” Three coders will separately screen these units of analysis for inclusion or exclusion using the checklist aforementioned. The three coders will be the lead researcher and two other qualified qualitative researchers with a media background and must have at least a master’s degree. In cases of doubt or disagreement, we will discuss the articles to reach a consensus about whether to include the news item based on the aforementioned criteria.

**Table 1:** Checklist for news items to be included in the study

Include	Yes	No
News item focusing on abortion	✓	
Aired on CITIZEN TV, NTV or KTN	✓	
Aired between January 1, 2016 and December 31, 2019	✓	
Social announcement regarding abortion		✓
Advertisement in regards to abortion		✓
News item merely mentioning abortion while focused on other topics		✓
Duplicate abortion coverage		✓

#### Data analysis

The selected news items will be transcribed and translated where necessary. Thereafter a coding frame will be developed to guide coders to make decisions in the analysis of the abortion news content. The coding frame will be piloted before we embark on the main analysis as described below.

The coding frame: A few televised abortion news transcripts will be selected from the larger sample of these as units of analysis. These transcripts will be selected equally from Citizen, KTN and NTV news items to reflect the full diversity of data sources. This will be followed by identification of the main categories and the subcategories for each main category [21]. This will be achieved by coding all the selected news items deductively where we will use existing literature and theory to develop some categories and subcategories [21] as well as inductively where the categories will be derived from the data [22]. This will be an iterative process and we will keep revising and refining the codes by combining some into larger categories and breaking some into subcategories [23].

Once the structure of the coding frame has been developed, we will provide a concise description of each category by stating what it means as well as the features and characteristic to this specific category [24]. Similarly, extensive definitions (a name, description, example and decision rules) of all the subcategories will be generated [24]. After the generation and definition of all the categories and subcategories, we will look at the structure of the coding frame once again and tighten any loose ends. At this stage, we will collapse subcategories that are very similar and conceptualize those that are more comprehensive as main categories [24]. We will refine categories and alter coding instructions until we are convinced that the coding frame we have will help us get the data we need.

The coding frame will therefore consist of category and subcategory names, definitions or rules for assigning codes, examples and an additional filed for taking notes as coding proceeds. The coding frame will be developed in Nvivo 11 where we will have the main categories, the subcategories and the coding units [24]. The sub-sample used in the code frame development will be used as part of the final dataset [25]. The coding frame will be piloted before we embark on the main analysis.

Piloting the coding frame: We will begin the piloting phase by selecting a sub-sample of the actual sample just like in the coding frame development described above. We will then conduct a trial coding whereby we will apply the categories from the coding frame to the selected material in a double-coded manner [26]. This means that all the three coders will be working independently of each other coding the transcripts in Nvivo 11 as described above. This procedure is similar to that which we will use during the main coding. Finally, we will check whether the units of coding will have been assigned to the same subcategories during double-coding.

Units of coding that will have been assigned to different categories and/or subcategories during the two rounds will be identified. Doubts and problems concerning the definitions of categories, coding rules, or categorization of specific cases will be discussed and resolved within the coding team [27]. These inconsistencies will reveal the subcategories that are difficult to use, those that can be used interchangeably and the overlaps between categories [24]. We will then revise the definitions of such categories and add decision rules where applicable in readiness for the main analysis. The coding frame will not be modified beyond this stage.

Main analysis phase: During the main analysis phase, we will begin by dividing the remaining part of the units of analysis equally among the three coders. Just like during the piloting phase, we will apply the categories from the coding frame to the selected material. However, unlike during the piloting, the transcripts will not be double-coded. Any coding inconsistencies will be discussed and resolved [24]. Supervisors will be consulted in the event that an inconsistency cannot be resolved.

### Objective 2: Explore Kenyan journalists’ perceptions and experiences with abortion framing in televised news media.

#### Study site

This study will also be conducted in Nairobi as the journalist working for the media houses targeted by this study are based in Nairobi.

#### Sampling

Criterion-based selection [28] will be used to select 12 journalists (9 reporters and 3 editors). Reporters will be selected based on their previous coverage of abortion stories on either CITIZEN TV, KTN or NTV television stations in the years January 2016 to December 2019. By-lines will be used to identify nine news reporters (3 Citizen TV, 3 KTN and 3 NTV) with the highest number of abortion news coverage. We will further purposively sample three news editors (1 Citizen TV, 1 KTN and 1 NTV) who have worked in the aforementioned television stations in the years January 2016 to December 2019.

#### Recruitment

The journalists (9 reporters and 3 editors) will be contacted and invited to participate in the study. A convenient date and time will be scheduled for the Key Informant Interviews.

#### Data collection

Key informant interviews will be conducted to understand how journalist (reporters and news editors) perceive their role in abortion coverage and their experiences with this coverage in the Kenyan landscape. Two interview guides (see Additional file 3) will be used for the KIIs; one for the reporters and one for the editors. The main domains of interest for the reporters interview guide will be experiences with health coverage, experiences with sexual and reproductive health and rights coverage, experiences with abortion coverage and experiences with adolescents’ abortion coverage. The main domains of interest for the editors’ interview guide will be experiences in their capacity as editors, experiences with sexual and reproductive health and rights coverage, experiences with abortion coverage and experiences with adolescents’ abortion coverage. The interviews will be conducted in a quiet and private place. Prior to beginning the KII, informed consent (see Additional file 1) will be obtained from each participant. Interviews will be conducted in either English or Swahili and will be audio-recorded. The participants will be reimbursed KES. 2000 ($ 20) as transport and meal allowance.

#### Data analysis

Data will be analyzed thematically. The analysis will involve six steps; transcription, familiarization, open coding, axial coding, categorization and report writing [29]. The audio-recorded interviews will be transcribed verbatim and translated where necessary. Thereafter the three coders will read each transcript to ensure that it makes sense, to obtain the general sense of information, and to reflect on the overall meaning of the data. The transcripts will then be exported to Nvivo® 11, a qualitative data analysis computer software package. Working together, the coders will open the transcripts in Nvivo® 11, examine each transcript highlighting, labeling and grouping together chunks that talked about distinct issues in relation to this study. Thereafter, the coders will explore the relationship of the codes generated above and grouping similar codes together into categories [30]. The different categories will then be grouped into themes; these themes should correspond with the main domains as presented in the interview guide. Although the above five steps are distinct, the analysis is iterative going back and forth between steps 3 and 5. Finally, the findings will be presented form of a research chapter within my dissertation.

### Objective 3: Examine adolescents’ (18–24 years) experiences and perceptions of media’s framing of abortion on Kenyan televised news media.

#### Study site

The study will be conducted in Uasin Gishu County, which lies on the northern part of Rift Valley province, in Kenya. Specifically, the study will be carried out among Moi University students. Moi University is a public university that draws students from all over the country thus presenting us with an opportunity to get a representative population.

#### Sampling

Purposive sampling will be used to select an estimated 48 university-going adolescents. The inclusion criteria for the study population will be; fully registered university students at the time of the study, attending Moi University main campus, aged between 18 and 24 years old, have access to a TV, have interest in news have access to a computer that has a webcam and a microphone, and has access to a set of earphones.

#### Recruitment

The study will be publicized through students class WhatsApp groups at the Moi University main campus. The poster that will be sent to these WhatsApp groups will state the nature, content and use of the study. Interested students will be directed to send a text message to the researcher’s phone number stating their names, age, gender and the course that they are pursuing. These criteria will ensure diversity of disciplines, a reasonable gender balance, and that the students are from the main campus of the university. Approximately 48 students will be chosen and invited to participate in a group discussion with 8–12 other participants.

#### Data collection

Four peer-led Focus Group Discussions (FGDs) with 8–12 participants will be conducted to explore adolescents’ perceptions and experiences with abortion coverage (Table 2). Four adolescent research assistants (2 male, 2 female) will be recruited and trained on how to conduct FGDs. A psychologist will also be present during this training to train them on psychosocial issues. The FGDs will be stratified along age, gender and year of study as illustrated below.

**Table 2:** FGD distribution

	Gender	Age	Year of study
FGD1	Male	18–20 years	1st and 2nd years
FGD2	Male	21–24 years	3rd and 4th years
FGD3	Female	18–20 years	1st and 2nd years
FGD4	Female	21–24 years	3rd and 4th years

The FGDs will be conducted virtually via ZOOM and an interview guide (see Additional file 4) will be used to guide the discussion. The researcher will send out an invitation link to the participants in advance and they will login at the scheduled time. The sessions will begin with general questions on media coverage of adolescents sexual and reproductive health issues. This will be followed by a presentation (vignette) of selected news items focusing on abortion, after which the sessions will center on questions related to abortion coverage in relation to adolescents. The vignette will be incorporated in the FGDs because we are cognizant of the fact that some adolescents might have not been previously exposed to abortion coverage. Prior to beginning the FGDs, informed consent (see Additional file 2) will be obtained from each participant. The sessions will be conducted in either English or Swahili and will be video-recorded using the ZOOM recording feature. Participants will be reimbursed KES 300 (USD 3) as an inconvenience fee. The research assistants will hold a virtual debrief after each FGD session to co-create field notes [31].

#### Data analysis

Data will be analyzed thematically and will follow the same procedure as that of Objective 2 described above.

### Data management

All audio and written files will be de-identified using unique identifiers and stored securely. Written files will be transformed into electronic copies within a day of the KII or FGD. Transcripts sent out for translation will have no identifiable information included with them. Recordings, transcriptions and data documents will be stored digitally using a cloud-based storage that is password protection and encryption. Only the study team will have access to this data. Any information used to recruit and schedule appointments, such as first name, mobile numbers, or addresses will be destroyed following data collection. Original audio-recordings will be deleted of the recorders following data check. All publications and presentations will present de-identified data.

## Discussion

Through examining the media framing of abortion, findings from this study have potential to facilitate more critical reflection and discussions on (un)safe abortion especially among adolescents, with a probable effect on journalistic framing of abortion as well as on health and social policies around abortion. Exploring adolescents’ experiences and perceptions of abortion coverage in the Kenyan broadcast media will be crucial to understanding how adolescents interpret and experience these abortion discourses in their everyday life. Furthermore, interrogation of abortion coverage segregated along gender lines is key to understanding men, and particularly adolescent boys, perceptions and experiences with abortion coverage. This is an important, yet barely explored area, because as co-conceivers of pregnancies being aborted, men play a key role (directly or in-directly) in women’s abortion trajectories. Thus, findings from this study will inform the development of effective interventions tailored towards addressing unintended pregnancies, preventing unsafe abortion and promoting adolescents’ maternal health while factoring in the current legal and social-cultural contexts. The study findings will be informative to those who wish to develop media that could be used to promote safe abortion as well as advocate for sexual reproductive health rights, especially among adolescents.

Literature on portrayal of abortion in news media is limited in number and scope. Previous studies predominantly quantitative and majorly conducted in the USA assert a lack of theoretical and methodological diversity. This has led to limited comprehensive understanding of media framing of abortion messages [7]. Methodologically, scholars have called for less descriptive strategies and more qualitative approaches to framing analysis research in order to advance the theory of framing as a major concept within the field of communication [8, 9]. Studies have urged future researchers to take up the use of intersectional frameworks of analysis such as integrating content analysis with interviews (In-Depth Interviews, Focus Group Discussions, etc.) to further explore framing research [10]. Theoretically, scholars have observed that framing is a process that has most of its analytical power when studied as an integrated model [11]. This entails mapping the media and audience framing process (framing, frame building and framing effects) in an integrative approach. However, notably lacking in literature is the frame production process which ultimately affects the portrayal of abortion messages [12–14]. Suggested ways of achieving this is by conducting interviews with journalists and editors so as to explore factors that influence frame-building [7, 15].

This study seeks to lay groundwork for future studies in news media framing of abortion messages by moving beyond the traditional approach of only interrogating media items to further exploring framing from the perspectives of media consumers and investigations in the process behind production of abortion messages. This will be achieved by triangulating content analysis, focus group discussions and in-depth interviews. The will be a unique contribution to literature as the study will empirically assess the different parts of the framing model and present the findings as an integrated model. The study moves beyond investigating only sub-sets of the framing model. Additionally it will lay groundwork for future studies in framing of abortion messages in news media in Sub-Saharan Africa (SSA), a study area that is generally lacking in literature.

## Plans for dissemination of research findings

Findings from this study will be widely disseminated. This will include availing the dissertation at the University of Witwatersrand library, oral presentations at conferences and/or to relevant bodies, and publication of manuscripts in relevant journal(s).

## Supplementary Information

**Additional file 1.** Informed consent for KIIs.

**Additional file 2.** Informed consent for FGDs.

**Additional file 3.** Semi-Structured Interview Guide for KIIs.

**Additional file 4.** Semi-Structured Interview Guide for FGDs.

## Data Availability

Materials described in this paper pertain to the study protocol only and there are no raw data reported. The datasets will be collected and analyzed and can be made available from the corresponding author on reasonable request. Interview guides developed for this study protocol are included as Additional files 1, 2, 3, 4.

## Abbreviations

KII: Key Informant Interview
FGD: Focus Group Discussion
SSA: Sub-Saharan Africa
APHRC: African Population and Health Research Centre
ASRHR: Adolescent sexual reproductive health and rights
SRHR: Sexual reproductive health and rights
QCA: Qualitative Content Analysis
