# Supplementary material for: Exploring media framing of abortion content on Kenyan television: a qualitative study protocol
Source: Reprod Health. 2021 Jan 19;18:12. doi: 10.1186/s12978-021-01071-5 (PMC7814727; doi:10.1186/s12978-021-01071-5)
Supplement: Supplementary file 1 — Additional file 1. Informed consent for KIIs. [file 12978_2021_1071_MOESM1_ESM.docx]

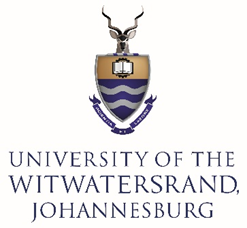


**Participant Information Sheet**

Dear Sir / Madam,

**Introduction:** My name is Catherine Kafu, a graduate student at the School of Literature, Language and Media, University of Witwatersrand. I am undertaking a PhD in Media Studies and as part of the fulfillment of my Degree; I am required to conduct research. My research is titled **‘Exploring media framing of abortion content on Kenyan television: A focus on adolescents’** under the supervision of Prof. Dina Ligaga and Dr. Juddy Wachira. You are invited to take part in this research study to help understand adolescents’ experiences with televised abortion coverage in Kenya. You were selected because you are aged between 18 and 24 years, and registered student at Moi University main campus at the time of the study. Carefully read this form and feel free to ask any questions you may have about the study.

**Purpose of the study:** The purpose of this study is to understand how abortion is framed in the televised news media in Kenya and the implications of these on adolescents’ perceptions and actions towards abortion. The findings will inform the development of effective interventions tailored towards addressing unintended pregnancies, preventing unsafe abortion and promoting adolescents’ maternal health while factoring in the current legal and social-cultural contexts.

**Number of people taking part in the study:** If you agree to take part in the study, you will be one of nine reporters or three editors in the study. Other participants include 48 university-going adolescents

**Procedures for the study:** If you agree to be in the study, we will protect your identity. We will ask you questions about your experiences with health coverage in general, your experiences with and role in abortion coverage with a focus on adolescent abortion coverage, your perception of the implications of abortion coverage and your recommendations on how to make abortion coverage more significant especially among adolescents. Interviews will be conducted in a private place of your choice by a trained researcher. The interviews will take about 30-60 minutes and will be audio recorded. Neither your name nor any other identifying information will be associated with the audio file or the transcript. Only the research team will be able to listen to the audio recording.

The audio files will be transcribed by the researcher and erased once the transcriptions are checked for accuracy. Transcripts of your interview may be reproduced in whole or in part for use in presentations or written products that result from this study. Neither your name nor any other identifying information (such as your voice) will be sued in presentations or in written products resulting from the study. Immediately following the interview, you will be given the opportunity to have the audio recording erased if you wish to withdraw your consent to audio recording or participation in this study.

**Risks of taking part in the study:** There are no major risks for being in the study. However, while on the study, you might experience some discomfort in answering some of the question. Additionally, there may be a risk of possible loss of confidentially. This risk will occur in case the researcher informs other persons about your unique study identification number, which may link you to the study. There we cannot assure complete confidentiality.

To minimize loss of confidentiality, any linking documents will be securely kept in a locked cabinet. No identifiable data will be associated with your study unique identifier. This unique identifier will be used on the data collection tools. In addition, data collection will be done in a quiet and private location of your choice.

**Benefits of taking part in the study:** There are no direct benefits to your participation. However, your contributions may be informative to those who wish to develop media that could be used to promote safe abortion as well as advocate for sexual reproductive health rights, especially among adolescents.

**Alternatives to taking part in the study:** There are no alternative procedures to this study.

**Confidentiality of records:** Your name or other information that may identify you will not be associated with your answers during this interview, the recording, transcription, or any other notes made by the note taker. We will transcribe the interview from the recording and destroy the audio file. The transcribed documents will be stored in secure locations and digital files will be protected with passwords. Only members of the study team will have access to the data.

**Dissemination:** The information you give me will be used to write a PhD dissertation that will be availed to the University of Witwatersrand Library. This information may also be used as a basis for articles and/or presentations in the future. However, your name or other information that may identify you will not be used in any publications or presentations

**Reimbursement:** You will not receive any form of payment for taking part in this study. However, you will receive KES. 2000 to cover your lunch and transport costs.

**Contact information:** For more information about the research, your rights as a participant, or any other concerns, please contact:

- The lead researcher, Catherine Kafu at 0725 243 852
- Moi University/Moi Teaching and Referral Hospital Institutional Research and Ethics Committee (IREC) on 0787 723 677
- National Commission for Science Technology and Innovation (NACOSTI) on 0735 404 245/ 0713 788 787

**Voluntary nature of study:** Your participation in this study is voluntary. You may refuse to participate and there will be no penalty for refusing. If you agree to participate, you may choose to discontinue your participation at any time or request that the information that you gave be withheld without penalty.

Yours sincerely,

Catherine Kafu,

Researcher

Tel: +254725243852

Email: [1540298@students.wits.ac.za](mailto:1540298@students.wits.ac.za)

**Consent Form**

In consideration of all of the above, I _______________________________________, agree to participate in this research study. The research has been explained to me and I understand what my participation will involve. I will be given a copy of this informed consent document to keep for my records. I agree to the following:

(Please circle the relevant options below).

| I agree that my participation will remain anonymous | YES NO |
| --- | --- |
| I agree that the researcher may use anonymous quotes in his / her research report | YES NO |
| I agree that the interview may be video recorded | YES NO |
| I agree that the information I provide may be used anonymously after this project has ended, for academic purposes by other researchers, subject to their own ethics clearance being obtained. | YES NO |

______________________________________________ (Signature)

______________________________________________ (Name of participant)

______________________________________________ (Date. *Must be dated by the participant*)

_______________________________________________ (Signature)

_______________________________________________ (Name of person seeking consent)

_______________________________________________ (Date)
