# Supplementary material for: Exploring media framing of abortion content on Kenyan television: a qualitative study protocol
Source: Reprod Health. 2021 Jan 19;18:12. doi: 10.1186/s12978-021-01071-5 (PMC7814727; doi:10.1186/s12978-021-01071-5)
Supplement: Supplementary file 3 — Additional file 3. Semi-Structured Interview Guide for KIIs. [file 12978_2021_1071_MOESM3_ESM.docx]

**INTERVIEW GUIDE (REPORTERS)**

**Introduction**

1. ***We will start by talking a little bit about you and your work as a reporter***
2. Tell me a little bit about yourself
   - - - 1. *Probe*: For how long have you been a reporter?
3. What type of journalism do you practice?

*Probe:* Do you have a beat bias? ***If no,*** why?

***If yes,*** which one? Why? For how long have you been focusing on this type of reporting?

1. **Experiences with abortion coverage**

***Now let us talk about your experience with abortion coverage***

1. Tell me about your experience with covering abortion stories

*Probe:* How often do you cover abortion stories? Why?

Which abortion issues have you majorly covered? Why?

What are some of the abortion stories that stood out the most for you? Why?

What specifically stood out for you in these stories? Why?

What was men involvement in the abortion coverage?

1. Who are mainly your sources of abortion stories? Why? How did you select these sources?

*Probe:* Are there any other sources that you would have wanted to use? Who? Why?

Why did you exclude them previously/why did you not work with them in previous

stories?

1. What/Who influences the abortion stories that you cover? How? Why?

*Probe:* Who decides which stories to air?

Who decides what angle to take with a given story?

Is there a time you have had to bin an abortion story? What was the story about? Why?

Who made the decision of binning the story?

Are there times you have had to make major changes (edit) on parts of an abortion

coverage? Which part(s)? Why?

1. What challenges have you encountered while covering abortion stories?

*Probe:* Pre-reporting period

Reporting period

Post-reporting period

1. What impact has your abortion coverage had?

*Probe:* Audiences (explore audience type)

Policy

1. **Experiences with adolescents abortion coverage**

***Let us focus on reporting about abortion among adolescents***

1. Have you ever covered adolescent abortion?

***If yes, probe:*** Tell me about your experience covering adolescent abortion

What aspects of abortion among adolescents have you covered?

What stood out the most for you during this coverage?

Who were your sources?

In your opinion, what factors influenced how you covered adolescent abortion? How? Why?

What challenges did you encounter while covering adolescent abortion?

If you were to do this coverage again, what would you do differently? Why?

What aspects of adolescent abortion would you want to shed more light on? Why?

Why do you cover abortion among adolescents?

What impact do you think this coverage has had on audiences? On policy?

What can be done to ensure that adolescent abortion coverage gets more prominence in

the broadcast media?

***If no, probe:*** Why have you never covered adolescents’ abortion?

What impact do you think such coverage would have on audiences? On policy?

What can be done to ensure that adolescent abortion coverage gets more prominence in the broadcast media?

**Recommendations for improving televised abortion coverage**

***Finally, let us focus on some of the things that can improve televised abortion coverage in Kenya***

1. Reflecting back on your experience with abortion coverage, what are some of the things that you would have done differently? Why?
2. What is lacking in abortion coverage in the Kenyan televised media?

*Probe:* What type of abortion messages would you want to see more often?

What sources would you want to see more often?

1. What are some of the things that can be done to ensure that abortion coverage gains more prominence in the televised media?
2. **THANK PARTICIPANT FOR THEIR TIME AND REASSURE THEM OF DATA CONFIDENTIALITY**

**INTERVIEW GUIDE (EDITORS)**

1. **Introduction and background information**

***We will start by talking a little bit about you and your work as an editor***

1. Tell me a little bit about yourself

***Probe***: -For how long have you been an editor?

-What do you do in a typical day at your workplace?

-How do you go about assigning reporter stories to cover?

-What factors influence the kind of stories you assign a reporter

-What informs your decision on what stories to air?

-What informs your decision to bin a story?

1. **Experiences with abortion coverage**

***Now let us move on to your experience with abortion coverage***

1. Generally, what has been your experience with abortion coverage?
2. How often do you assign reporters abortion stories to cover?/ How often do reporters come in with abortion footage?

Probe: -What factors influence your decision to assign reporters abortion stories? (Individual,

organizational, societal, national)

- What challenges have you encountered with abortion coverage during the pre-reporting period?

1. What/Who influences the abortion stories that you air? (Individual, organizational, societal, national) How? Why?

***Probe:*** -Who decides the angle that a given story takes?

-Tell me about a time when a reporter came back with an abortion story and you had to change the angle.

-Why did you change the angle?

-Is there a time you have had to bin an abortion story? What was the story about? Why? Who made the decision of binning the story?

-Are there times you have had to make major changes (edit) on parts of an abortion coverage? Which part(s)? Why?

1. Tell me about some of the abortion stories that stood out the most for you.

*Probe:* What specifically stood out for you in these stories? Why?

1. Who are some of the news sources that stand out for you during abortion coverage? Why?
2. What response/reaction have you gotten from abortion coverage? (Colleagues, Audiences, Authority)

*Probe:* How did you feel about this response? Why?/What was your reaction to this response?

1. What challenges have you encountered with abortion coverage during the post-reporting period?
2. **Experiences with adolescents abortion coverage**

***Let us now focus on coverage of abortion among adolescents***

1. Tell me about your experience with adolescent abortion coverage.
2. How often do you assign reporters adolescent abortion stories to cover?/ How often do reporters come in with adolescent abortion footage?
3. What factors influence your decision to assign reporters adolescent abortion stories? (Individual, organizational, societal, national)
4. What challenges have you encountered with adolescent abortion coverage during the pre-reporting period?
5. What aspects of adolescent abortion would you want reporters cover more? Why?

***For editors with no previous experience with adolescent abortion coverage***

1. Why have you never assigned reporters adolescents’ abortion stories to cover?

**Recommendations**

***Finally, as we wind up, let us focus on some of the things that can improve televised abortion coverage in Kenya***

1. In your opinion, what impact has abortion coverage had? (Probe: Audiences, Policy)
2. What would you have done differently with in terms of abortion coverage? Why?
3. What can be done to ensure that abortion coverage gains more prominence in the broadcast media? (Probe adolescent abortion coverage)

**THANK PARTICIPANT FOR THEIR TIME AND REASSURE THEM OF DATA CONFIDENTIALITY**
