# Supplementary material for: Exploring media framing of abortion content on Kenyan television: a qualitative study protocol
Source: Reprod Health. 2021 Jan 19;18:12. doi: 10.1186/s12978-021-01071-5 (PMC7814727; doi:10.1186/s12978-021-01071-5)
Supplement: Supplementary file 4 — Additional file 4. Semi-Structured Interview Guide for FGDs. [file 12978_2021_1071_MOESM4_ESM.docx]

**INTERVIEW GUIDE (FGD)**

**Study Title:** Exploring Media Framing of Abortion Content on Kenyan Television: A Focus on Adolescents

1. **Abortion generally**

***We will start by talking about abortion in general.***

1. What is your perception about abortion?
2. How do young people perceive abortion (probe: is this perception different from that of older people?)
3. Where do young people get information about abortion?

*Probe:* Which are the common platforms where abortion is discussed? {If they mention

media, probe for the exact type of media}

What are some of the things that are normally said about abortion on these platforms?

1. What are your thoughts about female involvement in abortion?
2. What are your thoughts about male involvement in abortion?
3. **Abortion in the media: Perception of news media portrayal of abortion**

***Now let us talk about abortion in the news media***

1. What kind of abortion messages have you heard/seen on the media?

*Probe:* How often do you hear these abortion messages in the media? {Mass media generally,

News media specifically}

Which are the most common messages? {Why do you think these messages are prominent in the news media?/ what makes these messages prominent in the news media}

To whom are these messages directed? Why?

1. Are there TV stations that have abortion coverage more often than others do?

*Probe:* Which TV stations?

Why do you think this is the case?

1. In your opinion, what impact has televised abortion coverage had?

*Probe:* On you as an individual, on young people, on the society

***Now we will move on to watching a 8:46 minute news feature that focuses on the experiences of the women who have undergone abortion, particularly those who do so in their teens. This feature was aired on KTN in 2018. It’s titled “Pregnancy and Abortion” and was done by Dr. Mercy Korir***

***Vignette***

***Reporter_Dr. Mercy Korir:*** *Mercy Atieno and Terry Kimani are friends living in the same neighborhood. Both are mothers. Each of one baby. Their friendship stems from a common story*

***Source 1_Female, Terry Kimani, Abortion Victim:*** *I always desired to pursue a Beauty and Hairdressing career but I fell out with my father and he no longer listens to me. He feels like I disappointed him in the past*

***Source 2_Female, Mercy Atieno, Abortion Victim:*** *The first time I procured an abortion was in 2014 after completing my studies. I was force to procure the abortion due to my parents’ situation, just thinking about the struggles that they were going through. I faced a lot of challenges throughout my educational journey. Additionally, the person who impregnated me did not to have anything to do with me. So I was forced to join a group of girls called MAGWANGE, these are girls who have no direction in life. These are girls who just roam around and try to live through life the best way they know. During my first pregnancy it was my friends who introduced me to the person who procures abortions, she was a traditional herbalist. When we went there they did not say that we were seeking to procure an abortion, they said we were seeking to clean the stomach. When you are pregnant and they give you that drug it cleans your stomach and also aborts the fetus*

***Source 1_Female, Terry Kimani, Abortion Victim:*** *When I was in form two I was impregnated by my boyfriend and after that he did not want to have anything to do with me. I was forced to abort because I don’t have anything so I can not afford to raise a child. I was about five months pregnant and I went to a pharmacy and talked to the attendant, he asked me to come back in the evening. I went back at around 9pm, he took me to the back room and inserted some medication in my vagina. He also gave me some oral drugs to take and then he asked me to go back home. He told me that the fetus will abort after about 12 hours. At around 6am I developed stomach cramps but the baby did not come out, that and the umbilical cord did not come out.*

***Source 2_Female, Mercy Atieno, Abortion Victim****I did not care because I was desperate, just thinking about the hardship that my parents were going through. My dad is a drunkard, seeing my mum’s struggles just forced me (to seek an abortion).*

***Source 1_Female, Terry Kimani, Abortion Victim:*** *It was in 2013 and I was in form two. I was forced to drop out of school because he impregnated me and after that he did not want to have anything to do with me. He said he wasn’t responsible for the pregnancy so I was just forced to…because I could nt raise a child single-handedly considering my situation at that time, I had nothing. That marked the end of my education, I also fell-out with my dad due to that. That experience changed me, I became very harsh and when they asked me to go back to school I refused. That was the time I joined that group, it’s a group of girls who go around doing nothing, they never miss any party. At that time I was in form two and I was at the peak of adolescence, I was not listening to anyone and I was not taking in any advice.*

***Source 2_Female, Mercy Atieno, Abortion Victim:*** *I procured a second abortion in 2016. In 2016 I procured two abortions because looking at how my mum was struggling I said ‘uhhh let us support each other (financially). Looking at my sibling, my mum’s struggles, my dad’s meager income that was insufficient, he use to leave us with 100 shillings, what is 100 shillings? [Amid Tears] So I used to engage in prostitution to support my mum. My mum also did not care where the money was coming from because I was supporting her. I remember my fourth abortion was in 2017 and that was the procedure that caused me problems; [still in tears] I thought I was going to die. My forth abortion almost killed me. I don’t remember whether she gave me medication…I don’t have a recollection of what happened. You know if you are used to something you can never imagine that it would go wrong. So when I took the medication the next thing I remember was being taken to FHOK while unconscious.*

***Source 1_Female, Terry Kimani, Abortion Victim:*** *I thought I was going to die. I will die we will both die. So I rushed to hospital.*

***Reporter_Dr. Mercy Korir:*** *Their plans to terminate pregnancies came at a cost*

***Source 1_Female, Terry Kimani, Abortion Victim:*** *My pregnancy was 5-6 months so he asked for seven thousand shillings. I was forced to sell my personal effects, the things that I brought back home with from school. Things like my dictionary, kamusi (Swahili dictionary), toughees (shoes), bag, and uniform to raise those funds.*

***Source 2_Female, Mercy Atieno, Abortion Victim:*** *She normally asks for one thousand shillings but if you negotiate she can charge you three hundred shillings and she does not know you she could take five hundred shillings. But for us we had become her regular clients so she used to charge us three hundred shillings (for the herbal medication). However, she is not aware of these girls intentions because we tell her ’I want medication for cleaning my stomach because I have been having stomach upsets’. So she gives it to you knowing that you have an upset stomach and that medication is meant to treat that problem. We used to use that medication wrongly. We knew that if we told her that it’s for abortion she would not give it to us.*

***Reporter_Dr. Mercy Korir:*** *These two young women are lucky to have survived their experience of terminating a pregnancy. They are unafraid to speak*

***Source 1_Female, Terry Kimani, Abortion Victim:*** *Let me tell you the truth, in this neighborhood when a girl gets pregnant and the man responsible for that pregnancy refuses to take up responsibility, the only option the girl thinks of is abortion. You will hear them say ‘let it be what it will be, it’s God who knows when you will die’. They will tell you ‘I will only die on the day that God planned, I have faith that God will protect me’*

***Source 2_Female, Mercy Atieno, Abortion Victim:*** *That is what most girls believe. They procure abortions at 6, 7, 8 weeks due to the low risk factor at during that period of pregnancy. Although there was a pregnancy that I terminated when it was still in the early stages to protect my image within my community. I told myself ‘let me terminate this pregnancy while it’s still in its early stages to avoid damaging my reputation’ although I did not have a reputation to uphold. Imagine terminating a pregnancy at five months when everyone knows that you are pregnant. When you procure an abortion, the fetus is disposed off in the sewerage, in the toilet.*

***Reporter_Dr. Mercy Korir:*** *This near death experience was not easy, it has it's fair share of weight on the girls conscience [06:59]*

***Source 2_Female, Mercy Atieno, Abortion Victim:*** *I just wanted to die because my reputation was already damaged among those who took me to hospital. I felt so guilty I asked God, ‘What kind of life is this that I am leading? Is it poverty that has turned me into this person who procures abortions and such like things? I hated myself for allowing men to exploit me sexuality because of money. At that time I did not have knowledge on family planning [amid tears]. I hated myself. I was even asking Aggrey ‘just let me die’ but he said no. He said ‘No. I want you to be a role model to girls like you who come here. I want to mentor you and you will in turn mentor them because if they hear from one or two people who have experienced it they will change’). We hold sessions with teenage girls aged fifteen to twenty and those aged twenty to twenty four. We normally tell them it’s possible to protect yourself from getting pregnant, but what about diseases. I urge them that as teenage girls if they must engage in sex they should use a condom. I further urge them to be very careful because men can not be trusted as they can prick the condom and if they can’t them they should just abstain. I tell them that it is better to abstain than to contract STIs such as gonorrhea. I am not afraid to tell them about my experiences, I normally tell them I messed up [tearing] and I wouldn’t want them to also go through what I experienced. I urge them to take up family planning. I tell them to take up a family planning method that suits them, ‘if the injection does not work for you, try another method’. I urge them to love themselves because these men are of no use to them, ‘they will get you pregnant and dump you’.*

***Reporter_Dr. Mercy Korir:*** *The family Health Options Kenya clinic in Kibra has been seeing the other side of abortions, the complications. They have found themselves offering post-abortion care which is more often than not, emergencies.*

*-END-*

1. What is your perception of this story?

*Probe:* Was this story portrayed positively or negatively? Why?

Do you think the media was comprehensive in how it covered this story?

How should this story have been portrayed?

1. Based on this clip, what is your perception of gender involvement in abortion coverage? (Probe female, male)

**Recommendations: Now let us talk about how you would want media to portray abortion messages**

1. What abortion messages do you feel have been missing in the news media?
2. What abortion messages do you want to see more often in the news media? Why?

*Probe:* Type of messages

Source of message

Persons these messages should be directed to

How should these messages be packaged

Media platforms where these messages should be aired

1. If you were given a chance to run a story on abortion aimed at young people in the mainstream media, what would you focus on?/what aspects of abortion would you focus on?/what messages would you focus on? Why?

*Probe:* Who would be your sources? Why

How would you package the message? Why?

What are some of the things you would do differently from what the current media is doing? Why? Which platforms would you use?

**THANK PARTICIPANTS FOR THEIR TIME AND REASSURE THEM OF DATA CONFIDENTIALITY**
